# Mature bony metaplasia in multinodular goiter: A case report

**DOI:** 10.1016/j.ijscr.2019.05.030

**Published:** 2019-06-05

**Authors:** Ali Al Khader, Esra Nsour, Anwar Alneweiri, Mohamad Al-Saghbini

**Affiliations:** a Faculty of Medicine, Al-Balqa Applied University, P.O. Box 19117, Al-Salt, Jordan; b Department of Pathology, Al Hussein Salt Hospital, Ministry of Health, Al-Salt, Jordan; c Al Hussein Salt Hospital, Ministry of Health, Al-Salt, Jordan; d Faculty of Medicine, Al-Balqa Applied University, Al-Salt, Jordan

**Keywords:** Bone, Osseous metaplasia, Thyroid

## Abstract

**Introduction:**

Various degenerative changes can be seen in multinodular goiter. These include cystic changes, hemorrhage, fibrosis and calcification. However, osseous metaplasia is extremely rare.

**Presentation of the case:**

Here we present a 44-year-old lady with multiple ill-defined thyroid nodules upon physical examination. Thyroid ultrasound showed multiple variably sized nodules with cystic degeneration. The largest left lobe nodule showed macrocalcification. Further evaluation was advised by the radiologist. Total thyroidectomy was performed and it revealed histological osseous metaplasia with lamellar bone formation. The clinical course following resection was unremarkable.

**Discussion:**

Osseous metaplasia with ectopic bone formation is extremely rare in benign thyroid disorders. To the best of our knowledge, only thirteen cases of sporadic goiter with heterotopic bone formation are reported.

**Conclusion:**

Osseous metaplasia can be a pitfall in the diagnosis of multinodular goiter. Ruling out comorbidities is mandatory, and further genetic and follow-up studies are needed.

## Highlights

• Osseous metaplasia with ectopic bone formation is extremely rare in benign thyroid disorders.
• Only thirteen cases of sporadic goiter with heterotopic bone formation are reported.
• Osseous metaplasia can be a pitfall in the diagnosis of multinodular goiter.
• Ruling out comorbidities is mandatory.

## 1 Introduction

Multinodular goiter is one of the most common surgical thyroid diseases. Moreover, thyroid nodules are seen in up to 85% of autopsy specimens [1]. The incidence increases with age and shows a significantly high female to male ratio [2]. The pathogenesis of multinodular goiter is still unknown. However, iodine deficiency, impaired hormone synthesis and increased insulin-like growth factor are among the suggested causes [3,4]. Various histopathological findings can be seen in thyroidectomies done for multinodular goiter. These include cystic changes, hemorrhage, fibrosis and calcification [5]. Osseous metaplasia with ectopic bone formation is extremely rare in benign thyroid disorders. To the best of our knowledge, only thirteen cases of sporadic goiter with heterotopic bone formation are reported [6,7]. In line with SCARE criteria, we present a 44-year-old lady with multinodular goiter showing histological osseous metaplasia and lamellar bone formation [8].

## 2 Report of the case

**Fig. 1:**
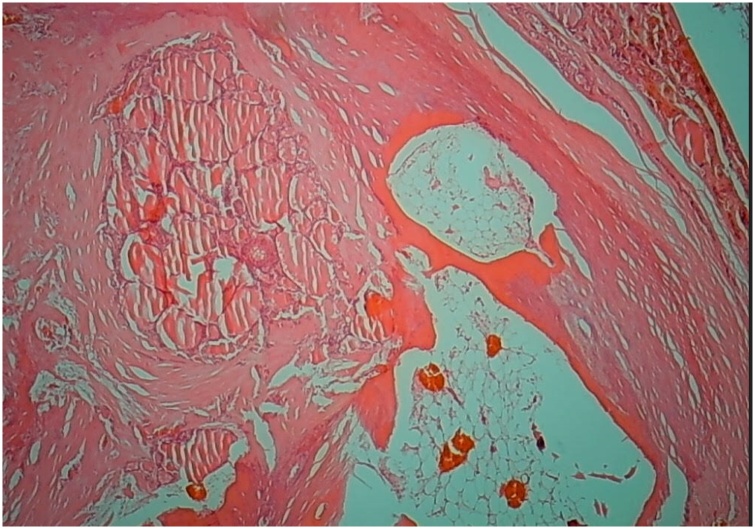
Lamellar bone with fatty marrow in a background of multinodular goiter and extensive intrathyroidal fibrosis (HE staining, ×40).

**Fig. 2:**
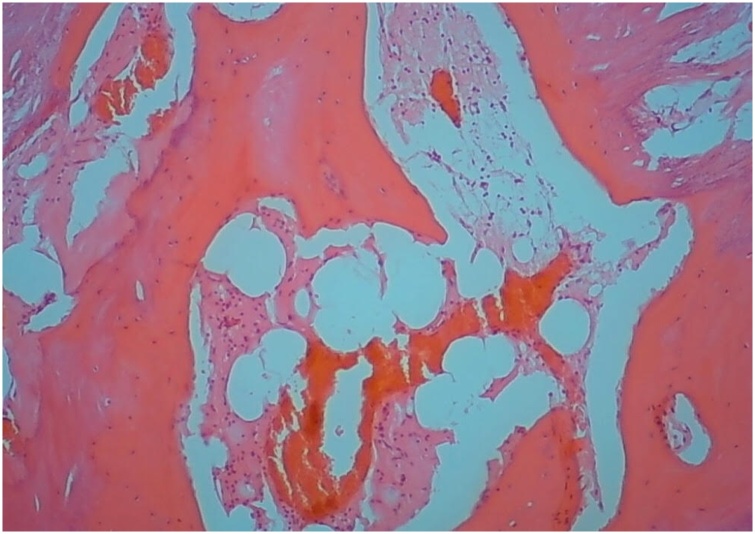
The lamellar bone contains osteocytes in lacunae and the fatty marrow is devoid of hematopoiesis (HE staining, ×100).

This 44-year-old lady had a 6-month history of thyroid enlargement. No compressive symptoms were reported. In addition, there were no symptoms of hyper- or hypothyroidism. The results of thyroid function test were normal. Physical examination revealed palpable ill-defined nodules, and thyroid ultrasound showed multiple variably sized nodules with cystic degeneration. The largest one in the right lobe measured 1.2 cm in maximum dimension, while the largest nodules in the left lobe and isthmus measured 0.6 cm and 1.1 cm, respectively. The largest left lobe nodule showed macrocalcification and further evaluation was advised by the radiologist. Total thyroidectomy was performed. Gross examination revealed multiple well-circumscribed nodules throughout the gland. Sectioning of the left lobe revealed a hard whitish mass that required decalcification. Microscopic examination of the hard nodule showed osseous metaplasia with lamellar bone formation and fatty marrow (Fig. 1, Fig. 2). This was surrounded by extensive fibrosis and nodular hyperplasia with focal cystic degeneration in the remaining thyroid tissue. The clinical course following resection was unremarkable.

## 3 Discussion

Multinodular goiter is the most common thyroid disorder with iodine deficiency being the main contributing factor [9]. A wide range of degenerative changes can accompany thyroid nodular hyperplasia. Of the changes commonly observed, dystrophic calcification is well-appreciated. However, osseous metaplasia with mature bone formation, as in our case, is extremely rare with only 13 cases reported in the English literature [6,7]. Our case was of an adult female and this was comparable to other reports [10]. Interestingly, all reported cases of mature bony metaplasia were of female patients. On the other hand, extramedullary hematopoiesis involving the thyroid was reported in 33 cases in the English literature with only 3 cases were of male patients [11]. The pathogenesis of this condition is still unknown. Bone morphogenetic proteins are of the major contributors in bone formation [12]. The initial steps in ossification require local osteogenic factors that induce the osteoblasts to synthesize collagens and ground substance. However, full maturation into lamellar bone requires an adequate concentration of calcium and phosphates that is necessary for the process of mineralization [13]. In one of the reported cases, a parathyroid abnormality was identified [14]. However, parathyroid glands were normal in our case. The presence of macrocalcification on the ultrasound in our case raised the suspicion that the calcified nodule may be malignant. This can be a diagnostic pitfall especially in the presence of osteoclasts in frozen section [6,15]. The extensive fibrosis present in our case was comparable to the cases reported by Handra-Luca et al. [6]. However, no inflammation was present and Riedel thyroiditis was excluded due to the absence of extrathyroid extension [16]. The thyroid function was normal in our case as documented in most of the reported cases. Only three reports documented hyperthyroidism [6,17,18].

## 4 Conclusion

In conclusion, osseous metaplasia can be a pitfall in the diagnosis of multinodular goiter. Ruling out comorbidities is mandatory, and further genetic and follow-up studies are needed.

## Conflict of interest

The authors declare that they have no conflict of interest.

## Sources of funding

This research did not receive any specific grant from funding agencies in the public, commercial, or not-for-profit sectors.

## Ethical approval

Not applicable.

## Consent

Written informed consent was obtained from the patient for publication of this case report and accompanying images.

## Author contribution

Ali Al Khader: Conceptualization, data curation, investigation, methodology, supervision, validation, visualization, Writing-original draft and Writing-review and editing.

Esra Nsour: Investigation, methodology, validation, Writing-original draft and Writing-review and editing.

Anwar Alneweiri: Data curation, investigation, methodology and Writing-review and editing.

Mohamad Al-Saghbini: Data curation, resources and Writing-review and editing.

## Registration of research studies

NA.

## Guarantor

Ali Al Khader.

## Provenance and peer review

Not commissioned, externally peer-reviewed.
